# Prognostic of different glomerular filtration rate formulas in patients receiving percutaneous coronary intervention: insights from a multicenter observational cohort

**DOI:** 10.1186/s12872-020-01621-y

**Published:** 2020-07-18

**Authors:** Wei Chen, Pengyuan Chen, Zhonghan Ni, Yuanhui Liu, Wei Guo, Lei Jiang, Xuebiao Wei, Jiyan Chen, Ning Tan, Pengcheng He, Yansong Guo

**Affiliations:** 1 Clinical College of Fujian Provincial Hospital, Fujian Provincial Hospital, Fujian Provincial Key Laboratory of Cardiovascular Disease, Fujian Provincial Center for Geriatrics, Fujian Medical University, Fujian Cardiovascular Institute, Fuzhou, 350001 China; 2 Department of Cardiology, Guangdong Provincial People’s Hospital’s Nanhai Hospital, The Second Hospital of Nanhai District Foshan City, Foshan, 528000 China; 3 Department of Cardiology, Guangdong Cardiovascular Institute, Guangdong Provincial Key Laboratory of Coronary Heart Disease Prevention, Guangdong Provincial People’s Hospital, Guangdong Academy of Medical Sciences, Guangzhou, 510100 China

**Keywords:** Non-ST elevation acute coronary syndrome, Percutaneous coronary intervention, Renal dysfunction, Prognosis

## Abstract

**Background:**

The relationships of renal dysfunction (RD) and chronic kidney disease (CKD) with prognosis have been well established among non-ST elevation acute coronary syndrome (NSTE-ACS) patients who receive percutaneous coronary intervention (PCI), but the efficacy of different estimated glomerular filtration rate (eGFR) formulas for predicting the prognosis is unknown.

**Methods:**

The cohort originated from a retrospective data, which consecutively enrolled 8197 patients. The eGFR was calculated by the Cockcroft-Gault, Modification of Diet in Renal Disease (MDRD), CKD Epidemiology Collaboration-creatinine, CKD Epidemiology Collaboration-Cys-C, CKD Epidemiology Collaboration-Cys-C-creatinine and a modified abbreviated MDRD (c-aGFR) equations in Chinese CKD patients. Patients were excluded if the eGFR could not be obtained by one of the formulas. Patients were categorized as having normal renal function, mild RD, moderate RD, severe RD, or kidney failure to compare prognosis. The primary outcome was the in-hospital net adverse clinical events (NACE). The secondary outcomes were NACE and all-cause death during follow-up.

**Results:**

In total, 2159 NSTE-ACS patients (age: 64.23 ± 10.25 years; males: 73.7%) were enrolled. 39 (1.8%) patients with in-hospital NACE were observed. During the 3.23 ± 1.55-year follow-up, 1.7% death and 4.2% NACE were observed in 1 year. The percentage of severe RD patients ranged from 15.4 to 39.2% according to different calculation formulas. A high prevalence of in-hospital NACE was observed in the severe RD groups (ranging from 8 to 14.3% for different formulas). Multiple regression analysis showed that a high eGFR is a protect factor against NACE and all-cause death regardless of the formula use. Receiver operating characteristic curves showed similar predictive performance of the c-aGFR when compared to other formulas (in-hospital NACE: AUC = 0.612, follow-up NACE: AUC = 0.622, and follow-up death: AUC = 0.711).

**Conclusions:**

Severe RD results in a high prevalence of in-hospital NACE in NSTE-ACS patients after PCI regardless of the formulas use. Different formulas have a similar ability to predict in-hospital and long-term prognosis in NSTE-ACS patients. The c-aGFR formula is the simplest and a more convenient formula for use in practice.

## Background

Renal function is a powerful predictor of short- and long-term outcomes in patients with acute coronary syndromes [1–3]. Studies have reported that 42.9% of patients with non-ST segment elevation acute coronary syndrome (NSTE-ACS) had concomitant chronic kidney disease (CKD) [4]. Therefore, in patients with acute coronary syndrome (ACS), the assessment of renal function is not only conducive to the judgment of the severity of kidney disease but also important for the prognosis. The estimated glomerular filtration rate (eGFR) is the clinical standard for the assessment of kidney function and provides a good overall evaluation of kidney function [5]. The currently available formulas for estimating the eGFR include the Cockcroft-Gault (CG), Modification of Diet in Renal Disease (MDRD), CKD Epidemiology Collaboration-creatinine (CKD-EPIcr), CKD Epidemiology Collaboration- Cystatin C (CKD-EPI_CysC_), CKD Epidemiology Collaboration-Cys-C - creatinine (CKD-EPIcr_-Cys-C_) and c-aGFR (a modified abbreviated MDRD equation based on the Chinese CKD patients) equations. Despite the consensus that renal dysfunction (RD) is a risk predictor of prognosis according to the latest NSTE-ACS guidelines [6], the clinical variation in different eGFR formulas remains unknown. However, no consensus exists regarding which eGFR formula is most suitable for predicting the prognosis of ACS [7–10].

The purpose of this study was to analyze the value of the five eGFR formulas (c-aGFR, CG, CKD-EPIcr, CKD-EPI_Cys-C_ and CKD-EPIcr_-Cys-C_) in predicting the prognosis of NSTE-ACS patients undergoing percutaneous coronary intervention (PCI).

## Methods

**Fig. 1:**
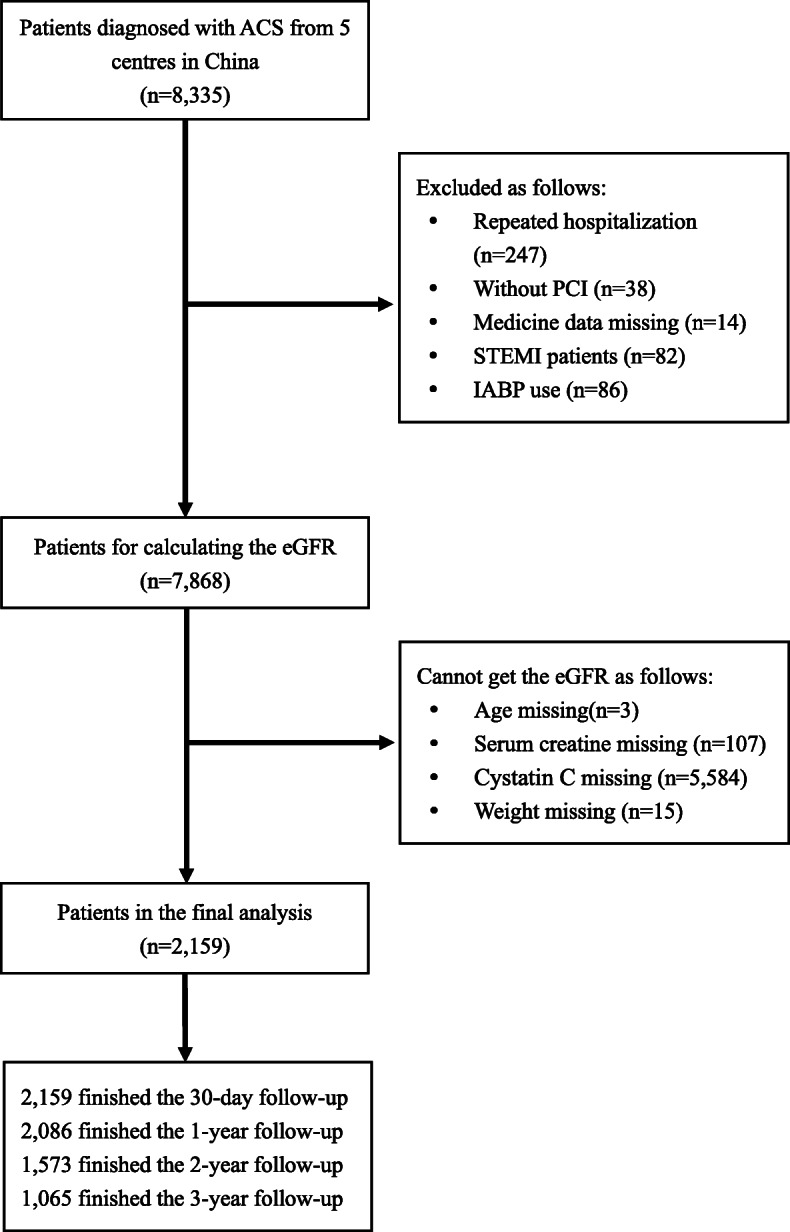
Flowchart of the study. ACS: acute coronary syndrome; PCI: percutaneous coronary intervention; STEMI: ST-segment elevation myocardial infarction; IABP: intra-aortic balloon pump; eGFR: estimated glomerular filtration rate

The present study originated from an observational cohort whose protocol were detailed in our previous study [11]. All 8197 patients who were recruited retrospectively from 5 centers in China were consecutively enrolled from January 1, 2010, to December 31, 2014. NSTE-ACS includes unstable angina pectoris (UA) and non-ST segment elevation myocardial infarction (NSTEMI). These definitions were consistent with our published study [11]. Patients for whom an eGFR could be calculated by the five formulas mentioned previously were included. The exclusion criteria were as follows: 1. patients who did not receive the PCI procedure, 2. patients who were readmitted, 3. ST-elevation myocardial infarction patients, 4. patients who were pregnant 5. patients with decompensated heart failure or cardiogenic shock who required intra-aortic balloon pump treatment. A total of 2158 patients were included in the final analysis (Fig. 1). All data were collected at the start of the study according to the predesigned form. The research program is consistent with the Helsinki Declaration and was approved by the local ethics committee.

Patients’ information was collected as we described in our previous study [11], and including preoperative creatinine level, age, sex, Cys-C, urea nitrogen, type of ACS (NSTEMI or UA), patient history, characteristic medications used during hospitalization, clinical presentation laboratory tests (NT-proBNP, peak troponin I, hemoglobin), and echocardiography. In-hospital, 30 day, 1-year and 3-year follow-up data were obtained by trained nurses through telephone interviews from November 7, 2015 to December 30, 2016. Relevant information was also collected from the clinical records of the residency registration system and from patients who were re-admitted.

We evaluated 5 formulas for assessing the eGFR based on creatinine and/or Cys-C levels as follows: c-aGFR, CG, CKD-EPIcr, CKD-EPI_Cys-C_ and CKD-EPIcr-_Cys-C_ (detail in supplemental materials). According to current Kidney Disease Improving Global Outcomes (KDIGO) guidelines, an eGFR of less than 60 ml/min/1.73 m^2^ was defined as significant RD [12]. Patients were categorized into 5 levels of renal function: normal (eGFR, > 90 ml/min per 1.73 m^2^), mild RD (eGFR, 60–90 ml/min per 1.73 m^2^), moderate RD (30–59 ml/min per 1.73 m^2^), severe RD (15–29 ml/min per 1.73 m^2^), or kidney failure (< 15 ml/min per 1.73 m^2^).

The primary endpoint was the in-hospital net adverse clinical events (NACE), which includes all-cause death, myocardial infarction and major bleeding (Bleeding Academic Research Consortium (BARC) score > 2,) [13]. the secondary outcomes were all-cause death as well as the NACE during the 1-year follow-up.

All data analyses were performed using SAS (version 9.4, SAS Institute, 210 Cary, North Carolina, USA), and a *P* value of less than 0.05 was considered as statistically significant. Categorical variables are expressed as counts (proportions), and differences among the groups were assessed using a chi-square test or Fisher’s exact probability method. Variables with a non-normal distribution are presented as the medians and IQRs. Continuous variables with a normal distribution are presented as the mean ± standard deviations. Differences among the groups were assessed using a t test or the Mann-Whitney U test for statistical analysis. Univariate analysis and multivariate logistic regression analyses were performed to calculate the adjusted odds ratios (ORs) and Hazard ratios (HRs) with 95% confidence intervals (CIs) for in-hospital mortality and NACE according to the eGFR (adjusted for sex, anemia, left main coronary artery stenosis, congestive heart failure, diagnosis (UA or NSTEMI), prior myocardial infarction (MI), prior stroke, stent number, stent length). The Kaplan–Meier method and log-rank test were used to analyze the all-cause mortality during the follow-up period. The area under each receiver operating characteristic curve (AUC) was calculated to assess the discriminative performance of each formula. All the missing data were eliminated.

## Results

Of all the 2159 NSTE-ACS patients, 39 (1.8%) patients with in-hospital NACE were observed. The clinical baseline characteristics of the patients between NACE and non-NACE are shown in Table 1. A total of 2159 patients were included in the study, of whom the mean age was 64.23 ± 10.25, 1591 (73.7%) were male. Patients with NACE were elder, have a higher rate of female, chronic heart failure and chronic kidney disease. They received more CCB, nitric acid, tirofiban and PCI stent. Notably, the serum creatinine and eGFR of the five formulas were all higher in the NACE patients’ group.

**Table 1:** Baseline characteristics between patients with NACE and non-NACE

Characteristic	Total (*n* = 2159)	non-NACE (*n* = 2120)	NACE (*n* = 39)	*P* value
Age (year)	64.23 ± 10.25	64.12 ± 10.26	70.15 ± 7.75	< 0.001
Age ≥ 65 years	1086 (50.3%)	1058 (49.9%)	28 (71.8%)	0.007
Weight (kg)	66.49 ± 12.14	66.55 ± 11.93	63.25 ± 20.55	0.323
Male sex	1591 (73.7%)	1569 (74.0%)	22 (56.4%)	0.013
Smoking	601 (27.8%)	590 (27.8%)	11 (28.2%)	0.959
CHF	274 (12.7%)	264 (12.5%)	10 (25.6%)	0.014
CKD	382 (17.7%)	370 (17.5%)	12 (30.8%)	0.031
MI history	342 (15.8%)	334 (15.8%)	8 (20.5%)	0.420
Prior PCI	400 (18.5%)	397 (18.7%)	3 (7.7%)	0.079
Prior CABG	22 (1.0%)	22 (1.0%)	0 (0.0%)	0.523
Stroke	160 (7.4%)	158 (7.5%)	2 (5.1%)	0.583
Atrial fibrillation	71 (3.3%)	70 (3.3%)	1 (2.6%)	0.798
Hypertension	1490 (69.0%)	1463 (69.0%)	27 (69.2%)	0.976
Diabetes mellitus	715 (33.1%)	698 (32.9%)	17 (43.6%)	0.301
Hyperlipemia	473 (21.9%)	462 (21.8%)	11 (28.2%)	0.337
Clopigrel or Ticagrelor	2139 (99.1%)	2100 (99.1%)	39 (100.0%)	0.553
Clopigrel/Ticagrelor loading	1422 (65.9%)	1398 (65.9%)	24 (61.5%)	0.565
Aspirin	2105 (97.6%)	2067 (97.6%)	38 (97.4%)	0.934
Aspirin loading	554 (25.7%)	534 (25.2%)	20 (51.3%)	< 0.001
Cilostazol	52 (2.4%)	50 (2.4%)	1 (2.6%)	0.007
Statin	2123 (98.5%)	2085 (98.4%)	38 (100.0%)	0.438
DAPT	2092 (97.0%)	2054 (97.0%)	38 (97.4%)	0.881
Warfarin	13 (0.6%)	13 (0.6%)	0 (0.0%)	0.624
ACEI/ARB	1650 (76.5%)	1622 (76.5%)	28 (71.8%)	0.488
CCB	544 (25.2%)	527 (24.9%)	17 (43.6%)	0.008
Nitric acid merged	1294 (60.0%)	1262 (59.6%)	32 (82.1%)	0.004
Beta blockers	1804 (83.6%)	1770 (83.5%)	34 (87.2%)	0.542
Tirofiban	204 (9.4%)	192 (9.1%)	12 (30.8%)	< 0.001
Number of stents	2 (1 ~ 3)	2 (1 ~ 3)	2 (1 ~ 3)	0.047
Total length of stents (mm)	39 (24 ~ 66)	38 (24 ~ 65)	51 (33 ~ 84)	0.023
Diagnosis				0.472
Unstable angina	1227 (56.9%)	1207 (57.0%)	20 (51.3%)	
NSTEMI	928 (43.1%)	909 (43.0%)	19 (48.7%)	
Cys-c (mg/L)	1.20 ± 1.77	1.19 ± 1.78	1.44 ± 0.98	0.138
Creatinine (μmol/l)	94.14 ± 69.35	93.47 ± 66.16	130.28 ± 166.34	0.176
Estimated GFR (ml/min/1.73 m^2^)				
c-aGFR	86.98 ± 29.53	87.19 ± 29.45	75.18 ± 32.21	0.012
CG	73.03 ± 27.93	73.31 ± 27.88	57.71 ± 26.58	0.001
CKD-EPIcr	77.18 ± 21.52	77.40 ± 21.41	65.37 ± 24.65	0.001
CKD-EPI_Cys-C_	71.72 ± 26.44	71.91 ± 26.36	61.23 ± 29.33	0.012
CKD-EPIcr_-Cys-C_	65.70 ± 21.18	65.90 ± 21.10	55.16 ± 23.13	0.002

*CHF* chronic heart failure, *CKD* chronic kidney disease, *MI* myocardial infarction, *PCI* percutaneous coronary intervention, *CABG* coronary artery bypass grafting, *DAPT* dual antiplatelet therapy, *ACEI/ARB* aldosterone receptor inhibitors and aldosterone receptor antagonist, *CCB* calcium channel blocker, *NSTEMI* non-ST segment elevation myocardial infarction, *eGFR* estimated glomerular filtration rate

The prevalence of significant RD, as identified by an eGFR of less than 60 ml/min/1.73 m^2^, ranged from a minimum of 15.4% using the c-aGFR formula to a maximum of 39.2% using the CKD-EPIcr_-Cys-C_ formula, with the other formulas providing intermediate values (CG 33%, CKD-EPIcr 20.4%, CKD-EPI_Cys-C_ 34.7%). The average eGFR values were 86.98 ± 29.53(c-aGFR), 73.03 ± 27.93(CG), 77.18 ± 21.52(CKD-EPIcr), 71.72 ± 26.44(CKD-EPI_Cys-C_), and 65.70 ± 21.18(CKD-EPIcr_-Cys-C_) ml / min / 1.73 m^2^ respectively (Table Appendix 1 in the supplemental materials).

**Fig. 2:**
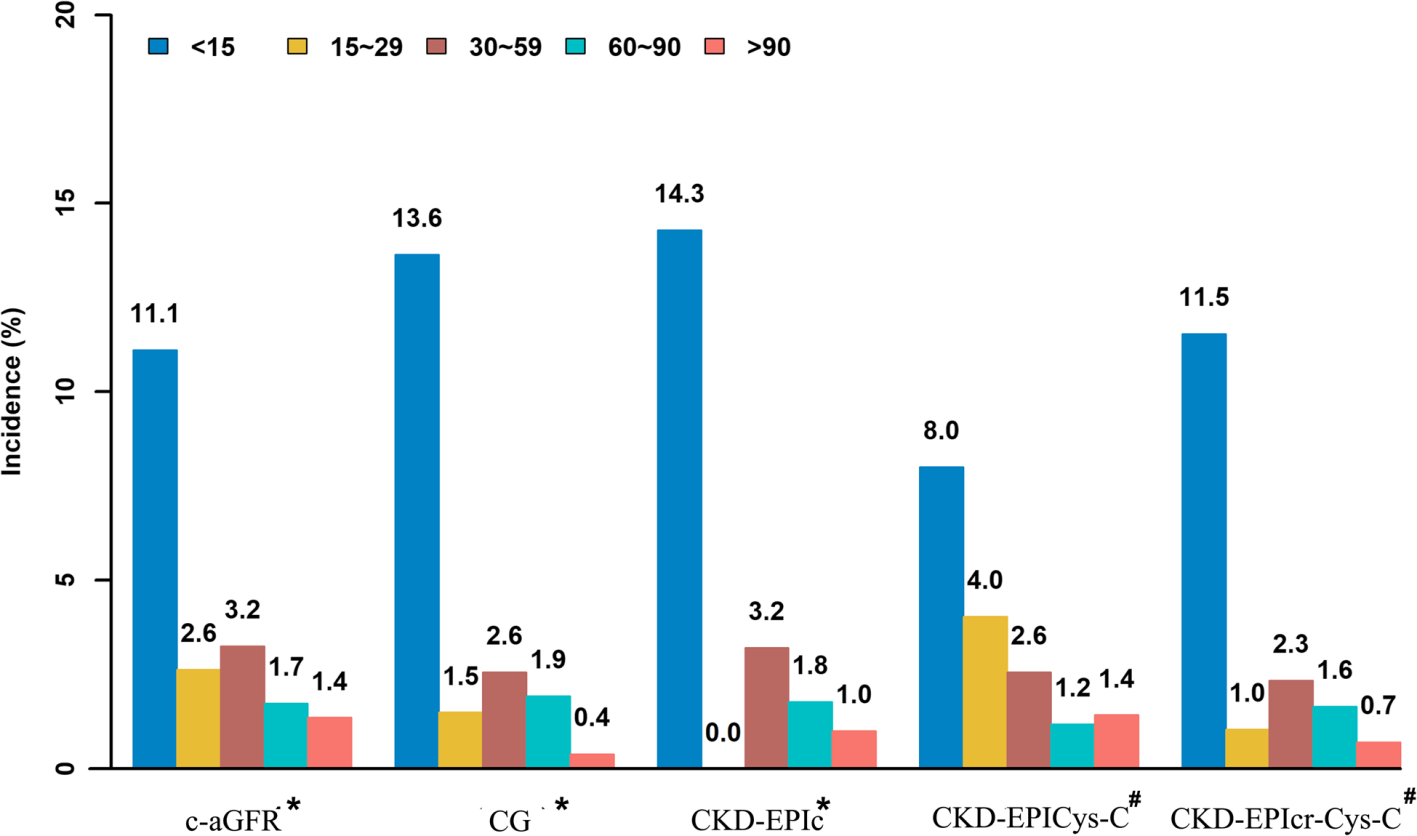
Incidence of in-hospital NACE based on renal function risk classification of different eGFR formulas. * indicates the *P* < 0.001; # indicates the *P* < 0.05

**Fig. 3:**
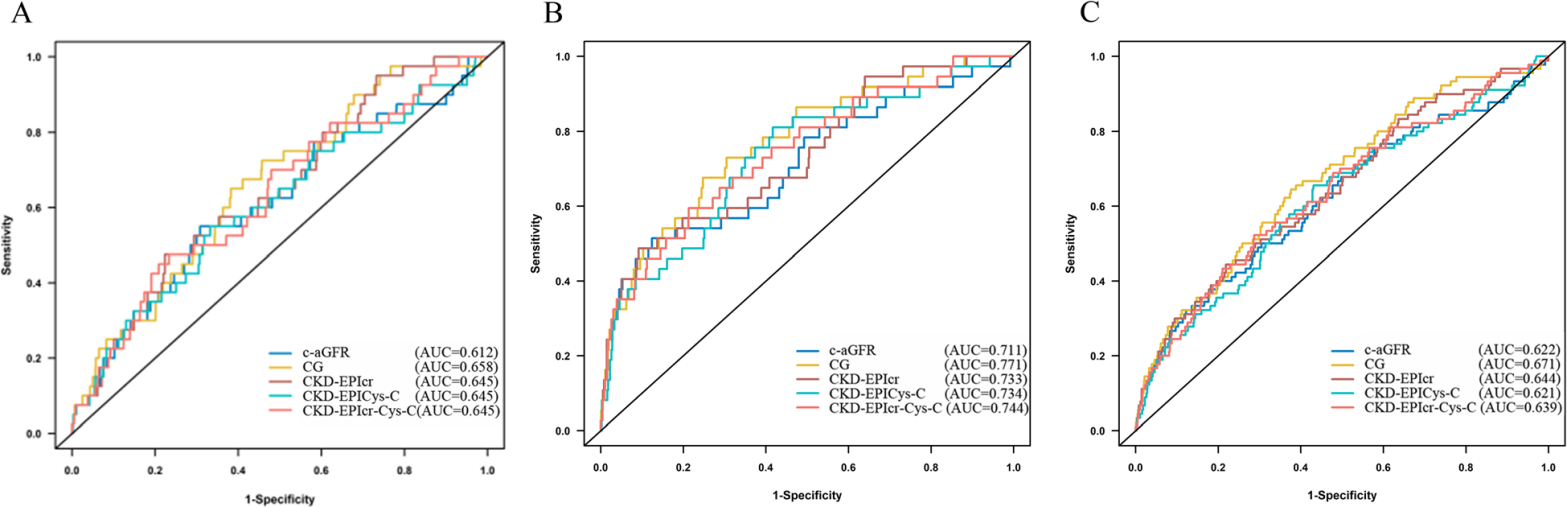
Receiver operating characteristic (ROC) curves for prognosis prediction using different eGFR equations. **a** the ROC curves for in-hospital NACE; **b** the ROC curves for 1-year NACE; **c** the ROC curves for 1-year death. AUC: area under the receiver operating characteristic curve; CI: confidence interval

The incidence rates of in-hospital NACE for different grades of renal function according to the various formulas are shown in Fig. 2 (Table Appendix 2 in the supplemental materials). Logistic regression analysis showed that a high eGFR is a protective factor against in-hospital NACE as well as follow-up NACE or death (Table 2). Five formulas showed the same discriminatory capacity for predicting NACE in the entire population, with AUC values of 0.612(c-aGFR), 0.658(CG), 0.645(CKD-EPIcr), 0.608(CKD-EPI_Cys-C_), and 0.630(CKD-EPIcr_-Cys-C_) respectively (Fig. 3a).

**Table 2:** Univariate and Multivariable Logistic or Cox Regression Risk Analysis for Prediction of Death and NACE

	Univariate		Multivariate	
	Odds or Hazard Ratio(95%CI)	*P* Value	Odds or Hazard Ratio(95%CI)	*P* Value
In hospital NACE				
c-aGFR	0.99 (0.97 ~ 1.00)	0.010	0.98 (0.97 ~ 1.00)	0.012
CG	0.98 (0.96 ~ 0.99)	< 0.001	0.98 (0.96 ~ 0.99)	0.002
CKD-EPIcr	0.98 (0.97 ~ 0.99)	0.001	0.98 (0.96 ~ 0.99)	0.002
CKD-EPI_Cys-C_	0.98 (0.97 ~ 1.00)	0.013	0.99 (0.97 ~ 1.00)	0.049
CKD-EPIcr_-Cys-C_	0.98 (0.96 ~ 0.99)	0.002	0.98 (0.96 ~ 0.99)	0.008
1-year NACE				
c-aGFR	0.98 (0.97 ~ 0.99)	< 0.001	0.99 (0.98 ~ 1.00)	0.004
CG	0.97 (0.96 ~ 0.98)	< 0.001	0.98 (0.97 ~ 0.99)	< 0.001
CKD-EPIcr	0.97 (0.96 ~ 0.98)	< 0.001	0.98 (0.97 ~ 0.99)	< 0.001
CKD-EPI_Cys-C_	0.98 (0.97 ~ 0.99)	< 0.001	0.99 (0.98 ~ 1.00)	0.012
CKD-EPIcr_-Cys-C_	0.97 (0.96 ~ 0.98)	< 0.001	0.98 (0.97 ~ 0.99)	< 0.001
1-year death				
c-aGFR	0.97 (0.96 ~ 0.98)	< 0.001	0.98 (0.97 ~ 0.99)	< 0.001
CG	0.97 (0.94 ~ 0.97)	< 0.001	0.97 (0.95 ~ 0.98)	< 0.001
CKD-EPIcr	0.96 (0.95 ~ 0.97)	< 0.001	0.97 (0.96 ~ 0.99)	< 0.001
CKD-EPI_Cys-C_	0.96 (0.95 ~ 0.98)	< 0.001	0.98 (0.96 ~ 0.99)	< 0.001
CKD-EPIcr-_Cys-C_	0.96 (0.94 ~ 0.97)	< 0.001	0.97 (0.95 ~ 0.98)	< 0.001

Abbreviations: *CI* confidence interval; Adjusted for sex, anemia, left main coronary artery stenosis, congestive heart failure, diagnosis (UA or NSTEMI), prior-MI and prior-stroke. All 5 equations were tested separately

**Fig. 4:**
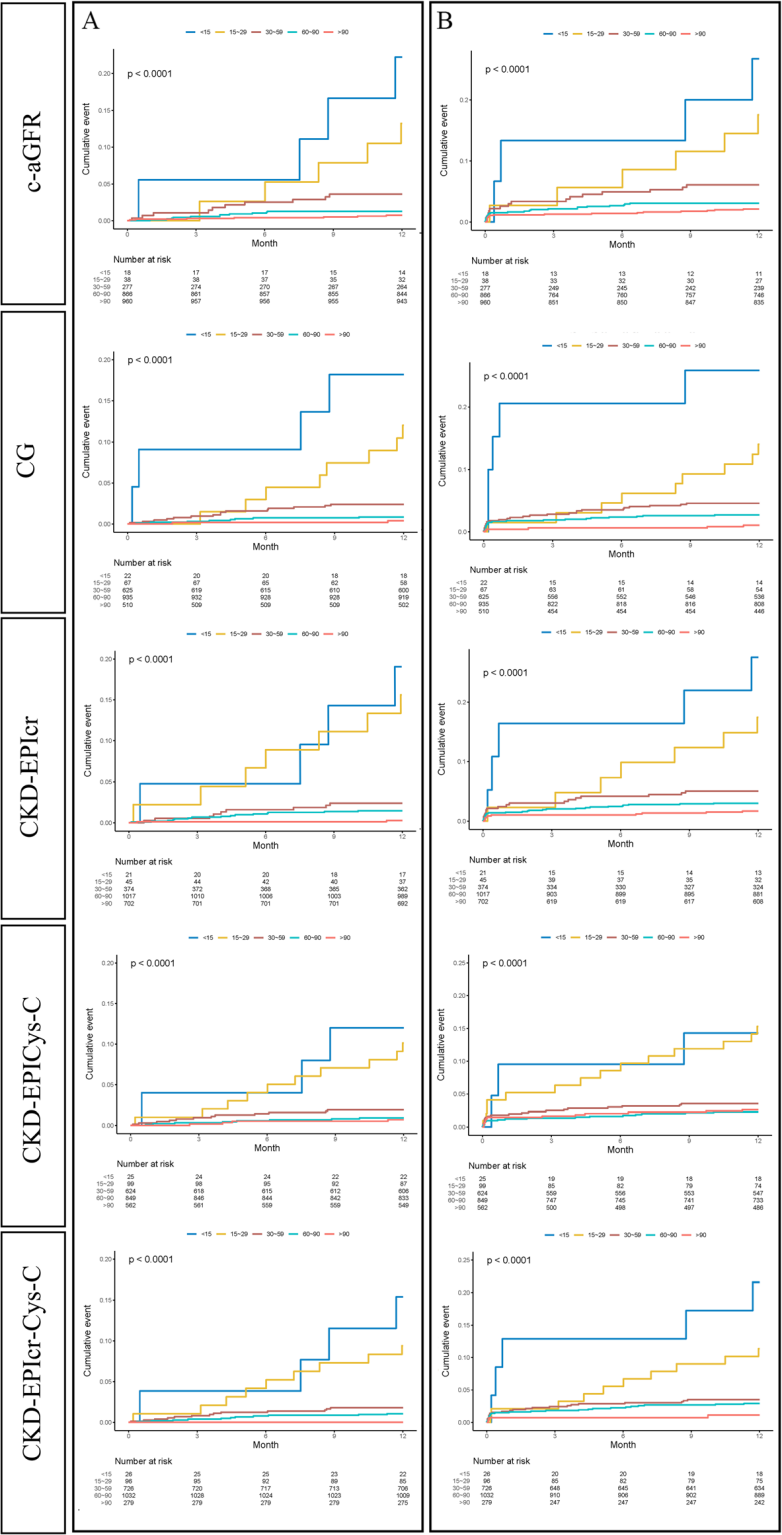
Kaplan-Meier survival curves for death (**a**) and NACE (**b**) within 1 year for different eGFR formulas

During the 3.23 ± 1.55-year follow-up, 1.7% death and 4.2% NACE were observed in 1 year. Kaplan-Meier survival analyses of death and NACE within 1 year categorized by renal function status according to the 5 different formulas are shown in Fig. 4. A total of 154 patients died during the 3 years of follow-up. All 5 formulas yielded similar trends in mortality and incidence rates. CKD staged by the 5 formulas was related to increased rates of mortality and NACE. The results of multivariable model and Cox regression fitting for the prediction of death and NACE using the various formulas are shown in Table 2. Regardless of the formula used, the risk of death for patients with a low glomerular filtration rate was always higher, and all formulas predicted death and secondary endpoints. The discriminatory power of each formula was assessed by calculating the AUC for the in-hospital NACE and death and NACE within 1 year (Fig. 3b and c).

## Discussion

This study showed the following results. First, although the glomerular filtration rates calculated by the five formulas were different and the proportion of each eGFR categorization varied markedly, the predictive value for the prognosis was consistent. Second, consistent with other studies, our study also found that RD is a good prognostic factor for NSTE-ACS patients undergoing PCI. Third, the c-aGFR formula, including only age and the serum creatinine level, is sufficient and practical for the prediction of eGFR compared to other formulas that include Cys-C.

Renal insufficiency is a predictor of coronary heart disease prognosis. However, there is no indicator in clinical work that can accurately reflect renal function, Including creatinine and Cys-C. When the kidney damage affects more than half of the entire kidney, serum creatinine will increase. The blood concentration of Cys-C is dependent on glomerular filtration without including any external factors, such as sex, age, or diet [14]. Cys-C is considered by many studies to be a relatively early and more sensitive marker than creatinine for indicating changes in renal function, but it is not measured in many hospitals. Creatinine clearance is the gold standard for assessing kidney function, but its detection is very complex and is only used for scientific research. Therefore, many evaluation formulas based on clinical indicators have been proposed. Renal function, which can be derived from different GFR evaluation formulas is considered a major risk factor for cardiovascular complications in patients with ACS [15].

Several studies have focused on assessing the prognostic value of eGFR formulas in different clinical situations. In the TRILOGY ACS Trial [2], Chiara Melloni et al. evaluated 8953 patients with ACS and found that worse CKD staging was significantly related to increased long-term risks of ischemic and bleeding outcomes. Conversely, in a recent registry study involving 1699 ACS patients, the CG equation presented a superior predictive ability for major adverse cardiovascular events to the MDRD-4 equation and was superior to the CKD-EPI equation [16]. Therefore, the difference between the predictive values of the CG formula and the CKD-EPI formula for prognosis showed mixed results.

Renal function is considered a major risk factor for cardiovascular complications in patients with ACS [15]. However, the conclusions of different studies vary regarding which formula has the best correlation with prognosis.

Piercarlo Ballo et al. followed 222 NSTE-ACS patients who underwent PCI, for 10 years to compare the predictive value of four creatinine-based eGFR formulas (CG, MDRD, CKD-EPI and Mayo-Quadratic). The Mayo-Quadratic formula was the best predictor of mortality, while the CKD-EPI formula showed the best performance in predicting cardiovascular events [7]. Katia Orvin et al. also suggested that the Mayo-Quadratic formula had better accuracy in predicting mortality than the MDRD, CG, CKD-EPI, and IB (an inulin clearance–based eGFR formula) formulas in patients with ACS [17]. The HOMAGE study, which used the CG formula adjusted for BSA, revealed a slight superiority in predicting cardiovascular mortality over the CKD-EPI and MDRD4 formulas in population with cardiovascular risk, heart failure and MI, but this advantage was not obvious in the general population [18]. Inês Almeida retrospectively assessed eGFR with the MDRD, CKD-EPIcreat, CKD-EPIcyst and CKD-EPIcreat/cyst formulas and suggested that the CKD-EPIcyst equation was superior to the MDRD formula for evaluating the mid-term mortality risk in patients admitted for ACS and added prognostic power to the GRACE score [19]. Conversely, in another larger sample size study, Axel Akerblom analyzed data from 13,632 patients in the PLATO study and found no difference in prognostic values for risk prediction of Cys-C- and creatinine-based formulas [17].

The simplified MDRD formula includes only 4 variables: sex, age, creatinine, and race; no difference in accuracy was found compared with other formulas recommended by the US CKD and dialysis clinical practice (K/DOQI) guidelines. The renal tubular secretion of creatinine differs in the Asian population, which exhibits differences in racial background and muscle mass. The national eGFR research team developed an improved original abbreviated MDRD formula (c-aGFR) based on the relevant data of 684 CKD patients in 2006, which showed smaller deviations and higher accuracy in Chinese CKD patients [20].

However, the current study focused on NSTE-ACS patients with PCI. These patients tended to be older, have more comorbidities (including worse renal function) and have worse long-term prognosis than ST segment elevation ACS patients [21]. Therefore, accurate long-term risk stratification of NSTE-ACS patients is even more crucial. Our study may therefore show that the five formulas have similar prognostic value for NSTE-ACS patients. Based on the original abbreviated MDRD formula, the c-aGFR formula is the simplest among the 5 formulas, can be easily used for the assessment of CKD patients [14] and is sufficient for assessing the prognosis of NSTE-ACS patients. Patients may not need to undergo Cys-C assessment as a routine clinical practice. Although a significant difference was observed between the CKD-EPI_Cys-C_ formula and combined CKD-EPI formula for predicting mortality, the range of superiority was limited. Additionally, according to our findings, the CKD-EPI_Cys-C_ formula may result in an overestimation of renal dysfunction, which does not affect the long-term prognosis.

### Limitations

There are some limitations of our research. First, our study is a retrospective cohort study with inherent limitations. Second, the estimation of GFR in this study is based on the creatinine value at admission. Importantly, creatinine may vary significantly in patients with ACS; therefore, the eGFR may be unstable. Third and notably, this study was conducted in NSTE-ACS patients undergoing PCI procedures, and therefore, our results cannot be extrapolated to patients who did not receive PCI. However, the PCI procedure has been recommended as a priority treatment among NSTE-ACS patients in the current guidelines. The current findings may be applicable to most current patients.

## Conclusion

Among NSTE-ACS patients who received PCI, those with severe RD exhibited a high prevalence of in-hospital NACE as well as follow-up NACE and death regardless of the formula used to calculate the eGFR. All five eGFR assessment formulas can be used to evaluate the prognosis of ACS patients and have similar predictive value. The c-aGFR, the simplest formula for assessing eGFR and is sufficient and practical for prognosis prediction in clinical work.

## Supplementary information

**Additional file 1.**

## Data Availability

The datasets used and analyzed during the current study are available from the corresponding author on reasonable request.

## Abbreviations

AUC: Area under the curve
BARC: Bleeding academic research consortium
c-aGFR: A modified abbreviated MDRD equation based on the Chinese CKD patients
CG: Cockcroft-Gault
Cis: 95% confidence intervals
CKD-EPIcr: CKD epidemiology collaboration-creatinine
CKD-EPICysC: CKD epidemiology collaboration- Cystatin C
CKD-EPIcr-Cys-C: CKD epidemiology collaboration-Cys-C creatinine
CKD: Chronic kidney disease
eGFR: Estimated glomerular filtration rate
HRs: Hazard ratios
K/DOQI: The US CKD and dialysis clinical practice guidelines
MDRD: Modification of diet in renal disease
NACE: Net adverse clinical events
NSTE-ACS: Non-ST elevation acute coronary syndrome
ORs: Odds ratios
PCI: Percutaneous coronary intervention
RD: Renal dysfunction
UA: Unstable angina pectoris
