# Supplementary material for: Prognostic of different glomerular filtration rate formulas in patients receiving percutaneous coronary intervention: insights from a multicenter observational cohort
Source: BMC Cardiovasc Disord. 2020 Jul 18;20:341. doi: 10.1186/s12872-020-01621-y (PMC7368721; doi:10.1186/s12872-020-01621-y)
Supplement: Supplementary file 1 — Additional file 1. [file 12872_2020_1621_MOESM1_ESM.docx]

**Supplemental materials**

**CONTENT**

[**Details of the 5 formulas** 2](#_Toc43636544)

[**Table appendix 1. Categorization of patients according to each eGFR equation.** 4](#_Toc43636545)

[**Table appendix 2 Distribution of NACE incidence in-hospital based on renal function status as determined eGFR according to the 5 different formulas.** 5](#_Toc43636546)

**Details of the 5 formulas**

(1) c-aGFR: Simplified MDRD formula (for Chinese populations) c-aGFR =175×SCr^-1.234^ ×age^-0.179^×0.79 (if female)

(2) CG: Cockcroft-Gault formula:

=(140-age) ×weight(kg)×0.85(if female)/(72×Scr)

(3) CKD-EPIcr: CKD-EPIcr formula：=141×(SCr/K)^a^×0.993^age^×1.018(if female)

K value: female=0.7, male=0.9

A value: female: SCr≤0.7, a=-0.329, SCr>0.7, a=-1.209

male: SCr≤0.9, a=-0.411, SCr>0.9, a=-1.209

(4) CKD-EPICys-C: CKD-EPI_Cys-C_ formula: =133×(Cys-C/0.8)^a^×0.996^age^×0.932(if female)

a: Cys-C ≤0.8 a=-0.499, Cys-C > 0.8 a=-1.328

(5) CKD-EPIcr-Cys-C: CKD-EPIcr_-Cys-C_ formula = 135×(SCr/k)^a^×(Cys-C/0.8)^b^×0.993^age^×0.969(if female)

k: female=0.7, male=0.9,

a: female SCr ≤ 0.7, a = -0.248; SCr>0.7 a=-0.601

male SCr ≤ 0.9, a= -0.207; SCr>0.9 a=-0.601

b: Cys-C≤0.8 mg/L b = -0.375; Cys-C>0.8; b= -0.711

Unified measurement units were used in the above 5 formulas to facilitate comparative analyses: Cr: mg/dl; Cys-C: mg/L; BSA: m^2^; age: years; body weight: kg; height: cm.

**Table appendix 1. Categorization of patients according to each eGFR equation.**

| eGFR equation | eGFR <15 ml/min/1.73 m^2^ | eGFR 15-29 ml/min/1.73 m^2^ | eGFR 30-59 ml/min/1.73 m^2^ | eGFR 59-90 ml/min/1.73 m^2^ | eGFR >90 ml/min/1.73 m^2^ |
| --- | --- | --- | --- | --- | --- |
| c-aGFR | 18(0.8%) | 38(1.8%) | 277(12.8%) | 866(40.1%) | 960(44.5%) |
| CG | 22(1.0%) | 67(3.1%) | 625(28.9%) | 935(43.3%) | 510(23.6%) |
| CKD-EPIcr | 21(1.0%) | 45(2.1%) | 374(17.3%) | 1017(47.1%) | 702(32.5%) |
| CKD-EPICys-C | 25(1.2%) | 99(4.6%) | 624(28.9%) | 849(39.3%) | 562(26.0%) |
| CKD-EPIcr-Cys-C | 26(1.2%) | 96(4.4%) | 726(33.6%) | 1032(47.8%) | 279(12.9%) |

**Table appendix 2 Distribution of NACE incidence in-hospital based on renal function status as determined eGFR according to the 5 different formulas.**

| eGFR equation | eGFR <15  ml/min/1.73 m^2^ | eGFR 15-29 ml/min/1.73 m^2^ | eGFR 30-59 ml/min/1.73 m^2^ | eGFR 59-90 ml/min/1.73 m^2^ | eGFR >90 ml/min/1.73 m^2^ | P value |
| --- | --- | --- | --- | --- | --- | --- |
| c-aGFR | 2(11.1%) | 1(2.6%) | 9(3.2%) | 15(1.7%) | 13(1.4%) | 0.011 |
| CG | 3(13.6%) | 1(1.5%) | 16(2.6%) | 18(1.9%) | 2(0.4%) | <.001 |
| CKD-EPIcr | 3(14.3%) | 0(0.0%) | 12(3.2%) | 18(1.8%) | 7(1.0%) | <.001 |
| CKD-EPICys-C | 2(8.0%) | 4(4.0%) | 16(2.6%) | 10(1.2%) | 8(1.4%) | 0.016 |
| CKD-EPIcr-Cys-C | 3(11.5%) | 1(1.0%) | 17(2.3%) | 17(1.6%) | 2(0.7%) | 0.002 |
